# Building up complexity in structural biology studies

**DOI:** 10.1038/s41594-024-01324-4

**Published:** 2024-06

**Authors:** Eva Nogales

**Affiliations:** 1 Molecular and Cell Biology Department, University of California, Berkeley, Berkeley, CA, USA.; 2 Howard Hughes Medical Institute, Berkeley, CA, USA.; 3 Molecular Biophysics and Integrated Bioimaging, Lawrence Berkeley National Laboratory, Berkeley, CA, USA.

## Abstract

Macromolecules are involved in myriads of interactions that regulate their cellular function. While years of structural biology progress was built by reducing this complexity, a molecular understanding of biological processes requires the characterization of ever larger and more dynamic molecular assemblies. Cryo-electron microscopy is rising to this challenge.

As structural biologists, we are reductionists by nature. Our macromolecule of interest is pulled out of the cell to be visualized in detail and interrogated about its shape and conformational landscape. But it is safe to state that no cellular component ever functions in isolation. In some cases, a macromolecule forms part of a stable complex, whereas in others it interacts with more transient molecular partners. Often both are true. Pursuing these interactions, stable or transient, is critical to obtaining a mechanistic understanding of molecular function. Structural biologists are becoming increasingly empowered to expand the compositional complexity of their samples and, with it, the insight of their studies. This Comment is a brief, personal reflection on some of the opportunities and challenges that cryo-electron microscopy provides in the structural characterization of ever larger and more compositionally and conformationally complex samples.

## Cryo-EM perks

Single-particle cryo-electron microscopy (hereafter cryo-EM) emerged as a generally applicable structural technique capable of sufficient resolution to allow the generation of atomic models about a decade ago. Both the commercialization of direct electron detectors that dramatically increase the quality of the experimental images and the availability of software packages that make the most out of those improved images have contributed to the maturation of the technique and what has been called ‘the resolution revolution’^1^. These developments in cryo-EM opened the door to the study of macromolecular assemblies of ever-increasing complexity at useful resolutions (they also opened the door to the investigation of other challenging samples, such as integral membrane proteins, but I will concentrate on large assemblies here). To start, a sample of large molecular size is, in principle, a plus in cryo-EM data analysis, in which signal is built out of noisy images of the molecule through alignment that benefits from the greater amount of information present in larger complexes. Additionally, because cryo-EM does not require large quantities of sample, the production of large recombinant or endogenously purified complexes does not require scaling up to challenging amounts. In fact, because samples can be concentrated on EM grids using multiple strategies, it is possible to visualize samples available only in the tens of nanomolar range. This benefit regarding sample requirements ultimately means that even endogenous purification of rare complexes can be used for structure determination. Such endogenous material may have value in and of itself. Well-designed affinity purification strategies engaging a bona fide member of a complex can provide the structural biologist with a complex that more closely resembles what is present in the cell, compositional complexity included. Both this compositional heterogeneity, and the conformational heterogeneity that is expected from most large macromolecular assemblies, are not insurmountable hurdles in structure determination by cryo-EM but rather technical challenges that require attention and that ultimately may provide extremely useful biological insight.

## Gains and challenges of endogenous complexes

Purification of endogenous complexes with the purpose of structural characterization makes little sense in the context of nuclear magnetic resonance, while for X-ray crystallography it is a practical approach only for a few types of samples, such as ribosomes (likely the most abundant large complex in prokaryotic cells and eukaryotic cytosol). On the other hand, such purification methods have been used very successfully in many cryo-EM studies (for example, see refs. 2,3). Endogenous complexes may be excellent initial targets to ensure that you start with the most complete set of components and with a physiologically relevant set of post-translational modifications. But this gain does come at a cost. For one, the sample may be more compositionally complex that you bargained for, depending on the stringency of your purification scheme. Another, very general problem is that, after the structure(s) is solved, testing of mechanistic models via mutational analysis can be a big challenge. The advent of CRISPR technology provides a generally applicable way of tagging one of the components, at its chromosomal locus, for affinity purification^4^. This procedure, however, requires that the tag not affect complex assembly (or function, if the complex is essential for the survival of your cells), and therefore may require some optimization and controls. Having to do this for mutants that test functionality would quickly become a huge endeavor. Thus, an ideal approach may be to gain information on composition and structure from bona fide endogenous complexes, and later apply this information to guide reconstitution approaches that can then be used to test mechanistic models via deletions and more targeted mutational analysis.

## Complex reconstitutions

The experimental path toward understanding molecular and cellular function often requires that we expand the study of an initially well-defined sample, no matter how large, to pursue its often-regulated interaction with substrates and regulatory partners. Assembly of dynamic supracomplexes on DNA or chromatin are examples near and dear to this author’s heart^5-9^. As the number of partners increases, the number of possible compositional states does as well, and this requires attention when the image processing pipeline is being optimized. Tactics for making the analysis robust in such cases may include a bootstrap approach of building up assemblies one step at a time and getting sequentially larger structures that need to be consistent. The process may involve separating complexes via methods such as GRAFIX^10^, in which the mixture is crosslinked as it is separated in a glycerol gradient. Or it may instead rely on computational approaches to deal with the mixture of complexes via 3D classification. Often, a combination of several such strategies may be needed.

## Pleomorphic assemblies

Sometimes understanding cellular function at the structural level involves unique arrangements of components that defy traditional structure determination. An example this author grapples with concerns mixtures of cytoskeletal components, in which filaments may be arranged in high-order, pleomorphic arrays by active interacting partners. Such samples are beyond the reach of cryo-EM in its single-particle implementation and require strategies that do not rely on obtaining 3D densities from the averaging of identical objects. In fact, this type of study is reminiscent of the challenges faced when attempting visualization of samples in situ, in the cellular context, with the exceptions of a simpler sample preparation (blotting versus focused ion bean milling) and an a priori knowledge of the full list of components. In such cases, cryo-electron tomography (cryo-ET), likely followed by sub-tomogram classification and averaging or a 2D template matching approach, is likely to be needed to define architecture and/or localize the different components (for a recent review on the interplay between single-particle cryo-EM and cryo-ET, in situ and in vitro, see ref. 11).

## Role of structure prediction and outlook

Structural studies of large, dynamic systems are nicely complemented by the powerful structure-prediction approaches that have proven so extremely effective in producing initial structures of proteins or multimeric assemblies^12,13^. The latter can inform the researcher both upfront, by defining possible interacting molecular partners, and at later stages, during the interpretation of experimental density maps, by providing starting models for all or parts of those maps.

Combining experimental and computational methods is both widening the scope of the systems that can be studied and accelerating the process of structure determination and the arrival of the resulting biological insights. As a consequence of these technical advances, the complexity of the samples that structural biologists can now consider for their studies is growing and will continue to do so, while in the process, the sophistication level and reach of their questions come closer to addressing the complexity that governs the regulated function of cellular components in their natural environment.
